# Supplementary material for: Improved AIS data simplification algorithm for extracting typical routes considering motion continuity
Source: PLoS One. 2026 Mar 11;21(3):e0340021. doi: 10.1371/journal.pone.0340021 (PMC12978504; doi:10.1371/journal.pone.0340021)
Supplement: Attachment — Submitted filename: Response to reviewers.docx [file pone.0340021.s001.docx]

### Dear Editor and Reviewers,

### Thank you for your thorough evaluation of our manuscript "Improved AIS Data Simplification Algorithm for Extracting Typical Routes Considering Motion Continuity". We have carefully addressed all major concerns through additional experiments and textual revisions. Our detailed responses are outlined below:

### **Reviewer 1:**

1. Please clarify the key differences between the proposed algorithm and representative methods such as Sliding Window, IOPW, and DTGO. In both the Introduction and lines Conclusion, highlight the specific practical problems your method can address that previous approaches could not.

Thank you for your suggestion. We have revised lines 25–32 in the Introduction, added corresponding statements in the Conclusion, and supplemented necessary references to clarify the key differences between our method and representative approaches. The revised manuscript now reads:

(1) Lines 25-32：**The extraction of typical routes thus holds notable practical significance, as it provides a concise reference for trajectory prediction\cite{ref10}, facilitates the detection of deviations thereby enhancing safety, supports efficient route planning, and enables the analysis of navigational strategies under diverse environmental scenarios\cite{ref11}. However, applying existing AIS data simplification algorithms, such as the Sliding Window (SW) and its variants (e.g., IOPW, DTGO), directly to this task of deriving typical routes from long-term cumulative data presents certain challenges. Their design typically assumes the presence of clearly defined start and end points for a single voyage, which may not be explicitly available in continuous, multi-voyage data streams. Furthermore, their performance often benefits from the availability of complete kinematic parameters, meaning their effectiveness can be diminished when such data is incomplete, as is common in practical AIS records. Most importantly, their core objective is to preserve the geometric shape of a single trajectory during compression, which differs from the goal of distilling a set of multiple, representative routes from a collection of overlapping voyages. Consequently, when applied to cumulative data, these methods may retain the inherent redundancy across numerous voyages rather than filtering it out to generate the concise set of typical routes.**
(2) Line 312: We have proposed an algorithm for converting AIS data into typical route data, leveraging distance threshold filtering and ship navigation patterns. **This approach is specifically tailored for extracting representative routes from cumulative data, a task that differs from the objective of traditional trajectory compression.** Our method begins with complex AIS data, applying a distance threshold to simplify the data and process it into multiple potential route segments. We then optimize these segments using ship navigation routes.
2. The mathematical derivations are not sufficiently systematic and contain logical gaps. For example, the choice of using twice the threshold (R_end = 2D_threshold + C) lacks theoretical justification.

Thank you for your suggestion. A geometrical and logical justification for the parameter R_end has been added following Eqs. (5) and (6).The revised manuscript now reads:

“**During the initial trajectory simplification, a new route segment is created when a point $p_{r}$ is beyond $D_{threshold} $ from its nearest point $p-{m}$ on an existing route, and $p-{m}$ is not the endpoint. This means the vessel's trajectory has diverged by a distance of at least $D_{threshold} $ to form a new branch.For an ending point of one segment to be considered for connection with another segment $R_{z}$, it must find a valid connection point on $R_{z}$. However, the point that caused the original divergence likely lies between $D_{threshold} $ and $ 2D_{threshold} $ from $R_{z}$. Therefore, setting $ R_{start} = 2D_{threshold}+C $ (where $C$ is a small constant to account for spherical distance calculation uncertainties) defines a search radius that is sufficiently large to encompass the potential divergence area and rediscover the original route connection point, yet constrained enough to avoid spurious connections with unrelated routes.**”

3. The dataset size and source are insufficient to demonstrate the generalizability of the algorithm. Using only 10 vessels’ AIS data is not representative.

Thank you for your suggestion. In this study, our primary goal was to propose and validate the core mechanics of the algorithm. Although the dataset comprises 10 vessels, it encompasses a volume of 420,000 records collected over five years. This long-term data captures a wide variety of navigational scenarios, including multiple routes, seasonal variations, and different operational patterns for each vessel. Consequently, the dataset possesses significant internal complexity and diversity, which we believe provides a robust foundation for demonstrating the feasibility and effectiveness of our proposed method. Furthermore, we fully acknowledge the value of further validation, and applying the algorithm to a broader range of real-world scenarios is a defined objective of our future work.

4. Provide the key hyperparameters of the deep learning models (e.g., number of layers, hidden units, learning rate, batch size, training epochs).

Thank you for your suggestion. We have revised lines 51–57 of the manuscript to explicitly highlight the key hyperparameters of the deep learning models. The revised manuscript now read:

“We applied three widely-used deep learning models for trajectory prediction, LSTM, GRU and Transformer, on both the raw AIS data and the simplified typical route data. The objective was to predict future vessel positions based on historical trajectory data. **For a consistent comparison, all models were implemented under a common framework. The LSTM and GRU models each comprised two recurrent layers with a hidden size of 256 units. The Transformer model was configured with a comparable capacity, featuring a 128-dimensional embedding size and 4 attention heads across its encoder-decoder architecture. All models were trained for 200 epochs using the Adam optimizer with a learning rate of 0.001 and a batch size of 64.**”

5. The explanations of figures (e.g., Fig. 4–6) are too brief, making it difficult for readers to fully understand their meaning.

Thank you for your suggestion. We have thoroughly revised the captions for Figures 4, 5, and 6 in the revised manuscript to provide a much more detailed and step-by-step explanation of their meaning and their specific roles in the algorithm.

6. The Discussion section mostly restates experimental results and lacks deeper reflection on the method’s limitations and potential directions for future work.

Thank you for your suggestion. We have added the method’s limitations and potential directions in Discussion section (after line 334). The revised manuscript now read:

“Through multiple rounds of experiments, we demonstrate that our algorithm is capable of processing trajectories formed from real AIS data, transforming these trajectories into typical routes for subsequent research use. **However, several limitations of our approach should be acknowledged. First, the performance of our algorithm is sensitive to the distance threshold parameter, whose optimal setting may require empirical tuning for different application scenarios. Second, our current method primarily relies on positional data and motion continuity, while other valuable AIS attributes such as vessel type and navigation status have not been fully utilized. These limitations point to promising directions for future work, including developing adaptive threshold selection methods and incorporating multi-dimensional AIS information to achieve more intelligent route extraction.**”

### **Reviewer 2:**

1. The core filtering parameter for trajectory simplification lacks explanation of how its value affects compression rate and route segmentation accuracy. Supplement experiments with different values to clarify the optimal parameter range.

Thank you for your suggestion. A geometrical and logical justification for the parameter R_end has been added following Eqs. (5) and (6).The revised manuscript now reads:

“**During the initial trajectory simplification, a new route segment is created when a point $p_{r}$ is beyond $D_{threshold} $ from its nearest point $p-{m}$ on an existing route, and $p-{m}$ is not the endpoint. This means the vessel's trajectory has diverged by a distance of at least $D_{threshold} $ to form a new branch.For an ending point of one segment to be considered for connection with another segment $R_{z}$, it must find a valid connection point on $R_{z}$. However, the point that caused the original divergence likely lies between $D_{threshold} $ and $ 2D_{threshold} $ from $R_{z}$. Therefore, setting $ R_{start} = 2D_{threshold}+C $ (where $C$ is a small constant to account for spherical distance calculation uncertainties) defines a search radius that is sufficiently large to encompass the potential divergence area and rediscover the original route connection point, yet constrained enough to avoid spurious connections with unrelated routes.**”

2. LSTM, GRU, and Transformer are used in trajectory prediction, but their hyperparameters are unspecified.

Thank you for your suggestion. We have revised lines 51–57 of the manuscript to explicitly highlight the key hyperparameters of the deep learning models. The revised manuscript now read:

“We applied three widely-used deep learning models for trajectory prediction, LSTM, GRU and Transformer, on both the raw AIS data and the simplified typical route data. The objective was to predict future vessel positions based on historical trajectory data. **For a consistent comparison, all models were implemented under a common framework. The LSTM and GRU models each comprised two recurrent layers with a hidden size of 256 units. The Transformer model was configured with a comparable capacity, featuring a 128-dimensional embedding size and 4 attention heads across its encoder-decoder architecture. All models were trained for 200 epochs using the Adam optimizer with a learning rate of 0.001 and a batch size of 64.**”

3. The two-step algorithm flow figure (e.g., Fig 3) lacks explanation of how "trajectory simplification"-output candidate routes link to the "trajectory optimization" module. Supplement captions or necessary notes.

Thank you for your suggestion. We have thoroughly revised the caption for Figure 3 in the revised manuscript to explicitly describe the two-stage workflow. The new caption now clearly states that the candidate routes output from the "Trajectory Simplification" module are the direct input to the "Trajectory Optimization" module, and it summarizes the key optimization procedures.

4. For route segmentation, the paper mentions distance-based judgment of route retention/addition but does not specify specific outcomes under different distance conditions. Add corresponding explanations.

Thank you for your suggestion.We have added explanatory text in line 140 (following Eq. (4)) that explicitly outlines the specific segmentation outcomes—creation of a new route, branching of a segment, or point retention—under different distance conditions, clarifying how the distance threshold governs the formation of candidate routes.. The revised manuscript now read:

“**The distance-based judgment in Eq.\ref{eq:4} yields distinct route segmentation outcomes depending on the spatial relationship between the current point $p_{r}$ and existing routes. When $D_{min} \ge D_{threshold}$ and the nearest point $p_{m}$ is the endpoint of its route ($ m=len(R_{z} $), a new route segment is created with $p_{r}$ as its start, capturing a true divergence. If $D_{min} \ge D_{threshold}$ but $p_{m}$ is not an endpoint ($m<len(R_{z})$), the algorithm branches a new segment from $p_{m}$, preserving alternative paths or shortcuts. Conversely, if $D_{min} < D_{threshold} $, the point $p_{r}$ is discarded to avoid redundancy, thereby maintaining a compact representation of the current route. This mechanism ensures that only significant deviations initiate new candidate routes.**”

5. Terms such as "starting point" and "initial waypoint" are used interchangeably. It is recommended to standardize the terminology, for instance, consistently using "starting point".

Thank you for your suggestion.We have thoroughly reviewed the entire manuscript and standardized the terminology related to the beginning of a route. The term "initial waypoint" and any other synonymous expressions have been consistently replaced with "starting point" throughout the text, including the methods, figures, and results sections.

6. Some references lack journal names or volume/issue numbers (e.g., Reference [18]). It is recommended to unify the reference format according to the journal's guidelines.

Thank you for your suggestion.We have carefully checked the reference list and completed the missing information (e.g., journal names, volume, and issue numbers) according to the journal's formatting guidelines. All references have been unified into the required format.

7. Other Minor Issues:
   Page 2: "We proposes" should be corrected to "We propose".
   Page 5: "p-m" should likely be "p_m".
   A thorough check for spelling and grammar throughout the manuscript is recommended.

Thank you for your suggestion.We have carefully checked the entire manuscript and corrected the identified errors, including "We proposes" to "We propose" on Page 2 and "p-m" to "p_m" on Page 5. A thorough proofreading has been conducted to eliminate spelling and grammatical errors throughout the manuscript.

We appreciate the reviewers' expertise in strengthening this study. Thank you for considering our work.

Best regards,

He Jin
